# Prognostic value of the ratio between right ventricular free wall longitudinal strain and systolic pulmonary artery pressure in patients with heart failure with reduced ejection fraction and ventricular secondary mitral regurgitation

**DOI:** 10.3389/fcvm.2025.1611772

**Published:** 2025-07-08

**Authors:** Călin-Dinu Hădăreanu, Diana-Ruxandra Hădăreanu, Despina-Manuela Toader, Maria-Livia Iovănescu, Cristina Florescu, Victor-Cornel Raicea, Ionuț Donoiu

**Affiliations:** ^1^ Doctoral School, University of Medicine and Pharmacy of Craiova, Craiova, Romania; ^2^ Department of Cardiovascular Surgery, Clinical Emergency County Hospital of Craiova, Craiova, Romania; ^3^ Department of Cardiology, University of Medicine and Pharmacy of Craiova, Craiova, Romania; ^4^ Department of Cardiology, Clinical Emergency County Hospital of Craiova, Craiova, Romania; ^5^ Department of Cardiology, Filantropia Clinical Hospital of Craiova, Craiova, Romania

**Keywords:** right ventricular to pulmonary artery coupling, right ventricular strain to pulmonary artery pressure, ventricular secondary mitral regurgitation, heart failure with reduced ejection fraction, prognosis, speckle-tracking echocardiography, three-dimensional echocardiography

## Abstract

**Background:**

In heart failure (HF) with reduced ejection fraction (HFrEF), ventricular secondary mitral regurgitation (V-sMR) leads to progressive impairment of right ventricular (RV) function and adversely affects outcomes. Non-invasive indices of RV–pulmonary artery (RVPA) coupling may offer enhanced prognostic value.

**Methods:**

We retrospectively evaluated advanced echocardiographic surrogates of RVPA coupling in 104 HFrEF patients with V-sMR.

**Results:**

Over a median follow-up of 526 days, 48 patients (46.2%) reached the composite endpoint of rehospitalization for HF decompensation or all-cause mortality. Patients who experienced events had significantly larger RV volumes, lower RV functional indices, and higher systolic pulmonary artery pressure (sPAP) compared with those without events. Among the RVPA coupling measures, the ratio of RV free-wall longitudinal strain (RVFWLS) to sPAP had the highest predictive accuracy (area under the curve 0.730), with an optimal cut-off of 0.46%/mmHg (71% sensitivity, 69% specificity). Kaplan–Meier analysis showed significantly lower event-free survival for patients with RVFWLS/sPAP < 0.46%/mmHg (log-rank *p* = 0.001). In multivariable Cox regression analysis, RVFWLS/sPAP (hazard ratio 0.110, 95% confidence interval 0.012–0.992; *p* = 0.049) emerged as an independent predictor of adverse outcomes.

**Conclusion:**

The RVFWLS/sPAP ratio, with a cut-off value of 0.46%/mmHg, is a robust, independent prognostic marker in HFrEF patients with V-sMR.

## 1 Introduction

Secondary mitral regurgitation (sMR) in patients with heart failure (HF) with reduced ejection fraction (HFrEF) represents a critical and multifactorial clinical challenge, and even mild sMR negatively impacts patient prognosis (1, 2). sMR contributes not only to left ventricular (LV) volume overload but also exacerbates pulmonary pressures, thereby increasing right ventricular (RV) afterload (3). On the other hand, sMR is a multifactorial condition influenced by LV dysfunction as well as by RV performance and pulmonary hemodynamics (4).

**Table 1:** Baseline clinical and echocardiographic characteristics stratified by event Status.

Parameter	Entire population (*n* = 104)	Events—(*n* = 56)	Events + (*n* = 48)	*P* value
Age (years)	69 [59–81]	72 [64–81]	65 [55–89]	0.089
Men (*n*, %)	78 (73%)	37	39	0.082
Atrial fibrillation (*n*, %)	28 (27%)	14	14	0.633
NYHA class III/IV (*n*, %)	61 (59%)	25	36	0.001
Type 2 Diabetes Mellitus (*n*, %)	33 (31.7%)	17	16	0.509
Arterial hypertension (*n*, %)	51 (49%)	29	22	0.551
Dyslipidemia (*n*, %)	55 (52.9%)	27	28	0.290
Coronary artery disease (*n*, %)	52 (50%)	29	23	0.773
Chronic kidney disease stage 4–5 (*n*, %)	13 (12.5%)	2	11	0.002
ACEi/ARNI (*n*, %)	79 (76%)	43	36	0.824
Betablockers (*n*, %)	89 (86%)	47	42	0.210
Spironolactone (*n*, %)	70 (67%)	42	28	0.125
Mitral regurgitation grade				0.010
Mild (*n*, %)	56 (54%)	36	20	
Moderate (*n*, %)	23 (22%)	13	10	
Severe (*n*, %)	25 (24%)	7	18	
Tricuspid regurgitation grade				0.346
None/Mild (*n*, %)	54 (52%)	10	6	
Moderate (*n*, %)	29 (28%)	23	15	
Severe (*n*, %)	21 (20%)	15	14	
3D LA maximum volume index (ml/m^2^)	56 [44–68]	56 [43–66]	56 [45–73]	0.593
LA reservoir strain (%)	9 [6–14]	10 [7–15]	8.5 [6–13]	0.242
LV global longitudinal strain (%)	7./5 [5.5–11.1]	8.3 [5.6–11.5]	6.5 [5–10]	0.068
3D LV EDV index (ml/m^2^)	123 [107–148]	121 [104–145]	126 [112–158]	0.088
3D LV ESV index (ml/m^2^)	80 [66–101]	80 [61–101]	82 [72–116]	0.169
3D LV SV index (ml/m^2^)	44 [38–49]	44 [36–49]	44 [39–53]	0.243
3D LV EF (%)	35 ± 8	35 ± 9	34 ± 8	0.328
sPAP (mmHg)	38 [28–45]	35 [26–44]	41 [33–57]	0.018
RV end-diastolic area index (cm^2^/m^2^)	12 ± 3	10 ± 3	13 ± 3	0.001
RV end-systolic area index (cm^2^/m^2^)	7.3 [5.3–10]	6.1 [4.7–7.6]	8.4 [6.4–11.7]	0.002
RV FAC (%)	38 [28–45]	40 [34–48]	33 [26–41]	0.008
TAPSE (mm)	17 [14–21]	18 [14–22]	16 [13–19]	0.098
RV FWLS (%)	17 ± 6	19 ± 6	15 ± 6	0.001
3D RV EDV index (ml/m^2^)	56 [40–82]	45 [34–64]	66 [47–85]	0.018
3D RV ESV index (ml/m^2^)	32 [20–46]	26 [17–38]	42 [26–49]	0.016
3D RV EF (%)	46 ± 10	49 ± 9	43 ± 10	0.006
2D RA maximum volume index (ml/m^2^)	28 [21–42]	28 [19–38]	29 [23–53]	0.067
RA reservoir strain (%)	19 [10–30]	25 [11–30]	17 [8–27]	0.148
3D RVEF/sPAP	1.19 [0.83–1.75]	1.43 [1.01–1.88]	0.96 [0.71–1.55]	0.003
RVFWLS/sPAP	0.43 [0.27–0.73]	0.53 [0.34–0.80]	0.31 [0.21–0.56]	0.001
RVFAC/sPAP	0.96 [0.63–1.45]	1.14 [0.79–1.65]	0.79 [0.49–1.08]	0.002
TAPSE/sPAP	0.44 [0.30–0.68]	0.54 [0.35–0.76]	0.39 [0.25–0.49]	0.011
3D RVSV/ESV	0.92 [0.58–1.10]	1.00 [0.68–1.10]	0.75 [0.52–1.14]	0.158

2D, two-dimensional; 3D, three-dimensional; ACEi, angiotensin-converting enzyme inhibitor; ARNI, angiotensin receptor-neprilysin inhibitor; EF, ejection fraction; ESV, end-systolic volume; FAC, fractional area change; LA, left atrium; LV, left ventricle; NYHA, New York heart association; RA, right atrium; RV, right ventricle; FWLS, free wall longitudinal strain; SV, stroke volume; sPAP, systolic pulmonary artery pressure; TAPSE, tricuspid annular plane systolic excursion.

Recent European guidelines on HF (1) and valvular heart disease (3) underscore the importance of comprehensive biventricular assessment in patients with sMR, with emphasis on the role of advanced echocardiographic techniques for both diagnosis and adequate risk stratification (5). Moreover, RV dysfunction by echocardiography robustly predicts the outcome in many cardiac conditions (6–8), including patients with sMR (9, 10). However, the classical, load-dependent RV functional parameters do not provide an accurate representation of RV intrinsic performance, because of the impact of loading conditions on RV function. In this context, echocardiography-derived surrogates of RV to pulmonary artery (RVPA) coupling expressed as RV functional parameters indexed to systolic pulmonary artery pressure (sPAP) estimated by Doppler echocardiography have emerged as indices of representation of RV systolic function in relation to afterload, and their prognostic value has been widely demonstrated (11–14). Furthermore, earlier studies focused on traditional measures such as the tricuspid annular plane systolic excursion (TAPSE)/sPAP ratio (15), that are limited by their load dependence and focus on only one aspect of RV mechanics. Recent advances in strain imaging and three-dimensional echocardiography allow for a more detailed and sensitive assessment of RV contractility, and three-dimensional echocardiography (3DE) is the method of choice when assessing RV size and function by echocardiography (16).

Accordingly, in this study we sought to determine the prognostic significance of both conventional and novel RVPA coupling parameters, derived from advanced echocardiography, in a conservatively treated HFrEF population encompassing the full spectrum of ventricular sMR (V-sMR) severity.

## 2 Methodology

**Figure 1:**
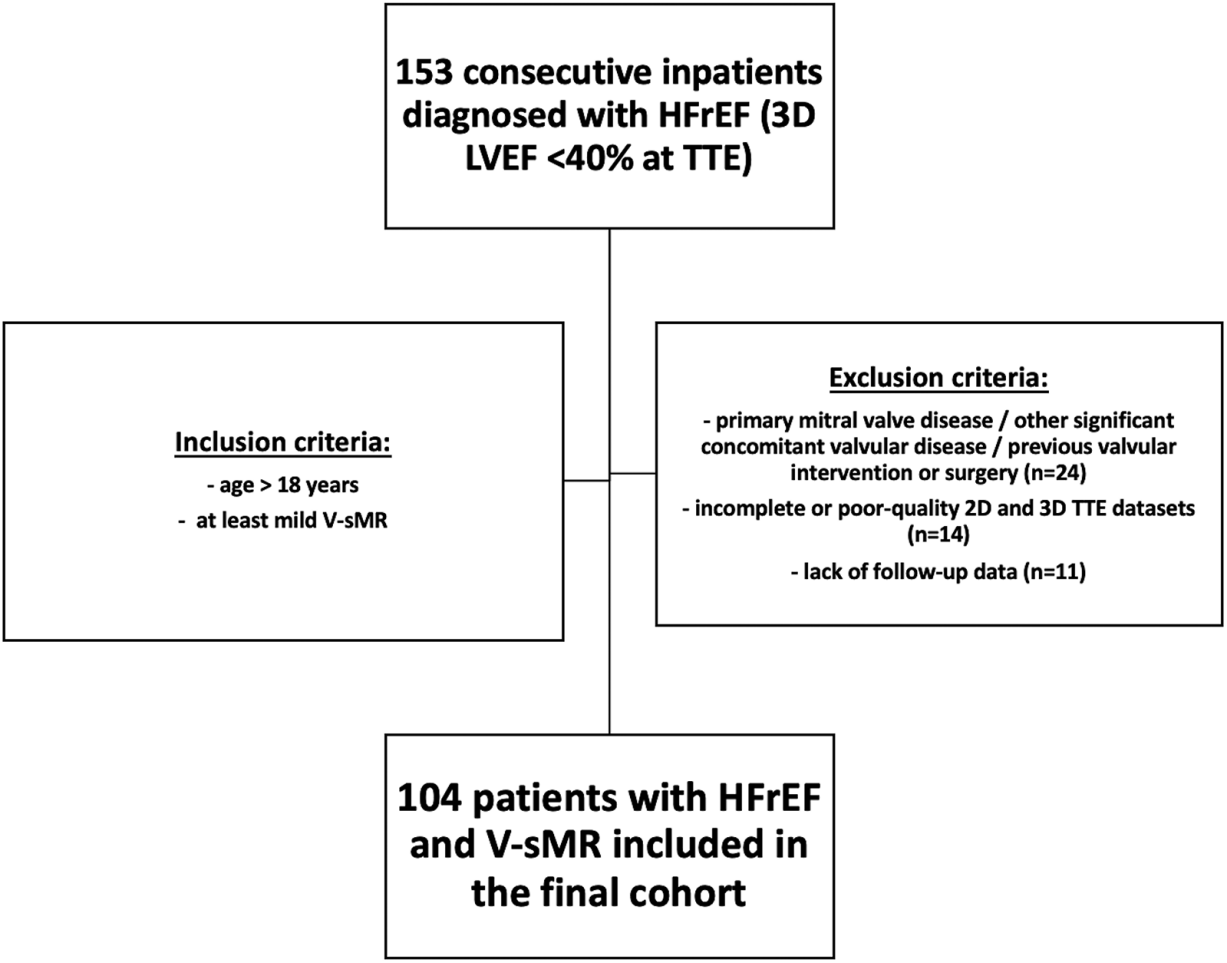
Flowchart describing patients’ selection process.

To fulfil our aims we have retrospectively analyzed the prospectively acquired, clinically-indicated transthoracic echocardiography (TTE) studies of inpatients diagnosed with HFrEF at the Clinical County Emergency Hospital of Craiova, Romania, a single tertiary center, between July 2020 and October 2022. The diagnosis of HFrEF was established according to current guidelines, and reduced EF was considered <40% at transthoracic 3DE. The inclusion criteria were (i) age > 18 years, (ii) a diagnosis of HFrEF, and (iii) at least mild V-sMR. The exclusion criteria were primary mitral valve disease, other significant concomitant valvular disease, previous valvular intervention/surgery, suboptimal acoustic windows precluding optimal image acquisition, incomplete TTE images, and the lack of follow-up data. The flowchart describing patients' selection process is presented in Figure 1. Demographic and clinical data were collected at baseline. The study was conducted in accordance with the Declaration of Helsinki, and approval of this study was obtained from the Ethics Committee of the University of Medicine and Pharmacy of Craiova (approval number 85/19.02.2024). Due to the retrospective nature of the study, the need for patients' informed consent was waived.

### 2.1 Echocardiographic acquisition and analysis

All participants underwent a comprehensive, clinically indicated advanced echocardiographic evaluation comprising two-dimensional (2DE), Doppler, speckle-tracking (STE) echocardiography and 3DE using commercially available Vivid E95 scanners (GE, Vingmed, Horten, Norway) equipped with 4Vc transthoracic probes. At the time of the TTE, patients were hemodynamically stable and had compensated HF following HFrEF treatment optimization. All examinations were conducted by experienced echocardiographers, and the digitally stored echocardiographic datasets were analyzed offline using EchoPAC version 204 (GE, Vingmed, Horten, Norway) by a single experienced operator who was blinded to the patients' medical histories. Conventional measurements of LV, RV, left atrial (LA), and right atrial (RA) size and function were obtained in accordance with current recommendations (16). SPAP was calculated from the maximal velocity of the TR jet and RA pressure estimate from inferior vena cava size and inspiratory collapsibility index (17). RV apical focused views were used for RA (18), RV FAC (19) and RVFWLS (20) quantification, and TAPSE by M-mode was calculated from the apical 4-chamber view (19). Advanced 3DE and speckle-tracking echocardiography (STE) (21, 22) analyses were performed using the dedicated software modules in EchoPAC v204 (4D AutoLVQ, 4D AutoRVQ, 4D AutoLAQ, and AFI LV, AFI RV, and AFI LA, respectively). Additionally, several non-invasive echocardiographic surrogates of RVPA coupling were derived as the ratios between RV functional parameters (TAPSE; fractional area change, FAC; RVFWLS, RVEF) and sPAP, as well as the ratio between RV end-systolic volume and RV stroke volume (RV ESV/SV). The severity of V-SMR, and tricuspid regurgitation (TR) was determined using a multiparametric algorithm, and the absence of structural mitral valve disease was verified by examining multiple cut planes derived from the volume-rendered 3DE dataset. While transesophageal echocardiography could provide additional morphological and functional insights of the mitral valve apparatus, particularly in complex cases, we opted to use transthoracic 3DE in our study, as clinically-indicated, especially since it is a reliable modality for evaluating MR when available (5). The severity of V-sMR was determined according to the effective regurgitant orifice area (EROA) measured by the proximal isovelocity surface area (PISA) method, using a threshold 30 mm^2^ to define severe V-sMR. All echocardiographic measurements were derived from the average of three consecutive cardiac cycles for patients in sinus rhythm or five consecutive cycles for those in atrial fibrillation. For the 3DE datasets, a minimum frame rate of 20 volumes per second was targeted. For STE measurements absolute values were used to facilitate the statistical analysis.

### 2.2 Reproducibility analysis

The intra- and inter-observer reproducibility of advanced echocardiographic measurements of RV volumes and function were assessed by calculating intraclass correlation coefficients (ICCs) and coefficients of variation (CVs). To evaluate intra-observer variability, a single researcher (D.R.H.) reanalyzed the same 15 randomly selected qualitative datasets while being blinded to the initial measurements. For inter-observer variability, the same datasets were analyzed by a different researcher (M.L.I.), who was unaware of the results from the first analysis.

### 2.3 Prognostic evaluation

The composite endpoint of the study was defined as a combination of rehospitalization for HF decompensation and all-cause mortality during the follow-up period. Data on patient survival and rehospitalizations were obtained through telephone interviews with patients or their family members and by reviewing electronic hospital admission records. Mortality status was independently verified using each patient's national identification number. For patients who did not experience any events, the date of their last recorded contact was used for survival analysis. Clinical events were rigorously adjudicated by physicians unaware of the patients' clinical and echocardiographic characteristics, based on predefined criteria in order to ensure consistency in the outcome determination.

### 2.4 Statistical analysis

Descriptive statistics were calculated for all variables, and the Shapiro–Wilk test was used to determine the distribution of continuous variables. Normally distributed continuous variables were reported as means ± standard deviations, whereas non-normally distributed variables were presented as medians with interquartile ranges (IQR). Categorical variables were expressed as frequencies and percentages. Group comparisons for continuous variables were conducted using Student's *t*-test for normally distributed data and the Mann–Whitney *U*-test for non-normally distributed data, while the chi-square test was used for categorical comparisons.

For event-free survival analysis, both Cox proportional hazards regression and Kaplan–Meier analyses were performed. In the univariable Cox regression model, variables with a *p*-value less than 0.05 were identified as potential predictors of the composite endpoint and subsequently included in the multivariable model, with results reported as hazard ratios (HR) and corresponding 95% confidence intervals (CI). Before including the variables in the multivariable model, the variance inflation factor was calculated to rule out multicollinearity, with values between 1 and 10 indicating no collinearity. Receiver operating characteristic (ROC) curve analysis was conducted to evaluate the predictive performance of the RVPA coupling surrogates, and the optimal cut-off was determined using Youden's J index. To assess the statistical significance of the difference in AUCs between RVPA coupling indices, the DeLong test was performed, The cut-off value derived from the ROC curve was then used to stratify patients in the Kaplan–Meier survival analysis, with survival curves compared using the log-rank test. A two-tailed *p*-value < 0.05 was considered statistically significant. All statistical analyses were carried out using SPSS version 23 (SPSS Inc., IBM Corp., Chicago, IL, USA), and R statistical software for Mac.

## 3 Results

The final study cohort (Table 1) comprised 104 patients with HFrEF and V-sMR (median age 69 years, 73% men), of which 59% in NYHA class III/IV. The average 3D LVEF was 35 ± 8%, with a median LV end-diastolic volume (EDV) indexed to body surface area of 123 [107–148] ml. The severity of V-sMR was distributed as 54% mild, 22% moderate, and 24% severe, while TR was mild in the majority of cases (52%).

During a median follow-up of 526 days, 48 (46.2%) patients met the composite outcome of rehospitalization for HF decompensation and all-cause mortality. The patients who reached the composite endpoint were more frequently in NYHA class III/IV (*p* = 0.001), had a higher prevalence of advanced (stage 4 and 5) chronic kidney disease (*p* = 0.002), and had more severe V-sMR. However, no statistically significant differences were found regarding LV and LA size and function between patients who reached the composite endpoint and those who did not.

Conversely, RV evaluation showed significant differences between groups. Patients with events had increased RV size, both in terms of areas (*p* = 0.001 for RV end-diastolic area index, and *p* = 0.002 for RV end-systolic area index), and 3D volumes (*p* = 0.018 for RV EDV index, and *p* = 0.016 for RV ESV index) compared to patients without events. Furthermore, they also had more dysfunctional RV (lower RV FAC, *p* = 0.008, RVFWLS, *p* = 0.001, and 3D RVEF, *p* = 0.006). Nonetheless, sPAP was higher in patients with events (*p* = 0.018), who also showed more severe RVPA uncoupling (lower values for all RVPA coupling surrogates, *p* < 0.05, except for RVSV/ESV).

**Figure 2:**
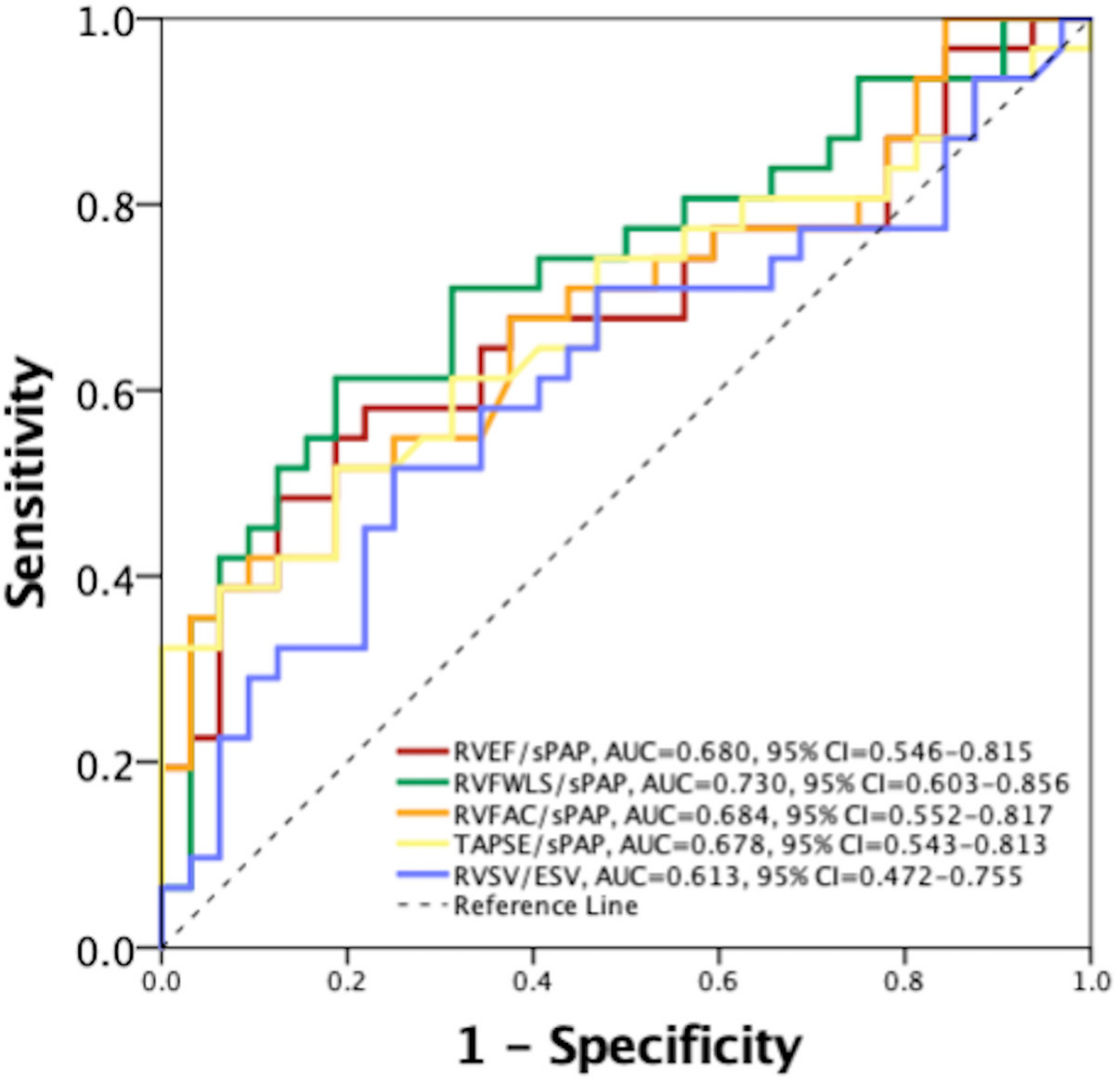
Receiver operating characteristic curve for comparison of the prognostic value between echocardiographic right ventricular to pulmonary artery coupling indices. AUC, area under the curve; CI, confidence interval; RVEF, right ventricular ejection fraction; RVFAC, right ventricular fractional area change; RVFWLS, right ventricular free wall longitudinal strain; RVSV/ESV, right ventricular stroke volume to end-systolic volume; sPAP, systolic pulmonary artery pressure; TAPSE, tricuspid annulus plane systolic excursion.

**Figure 3:**
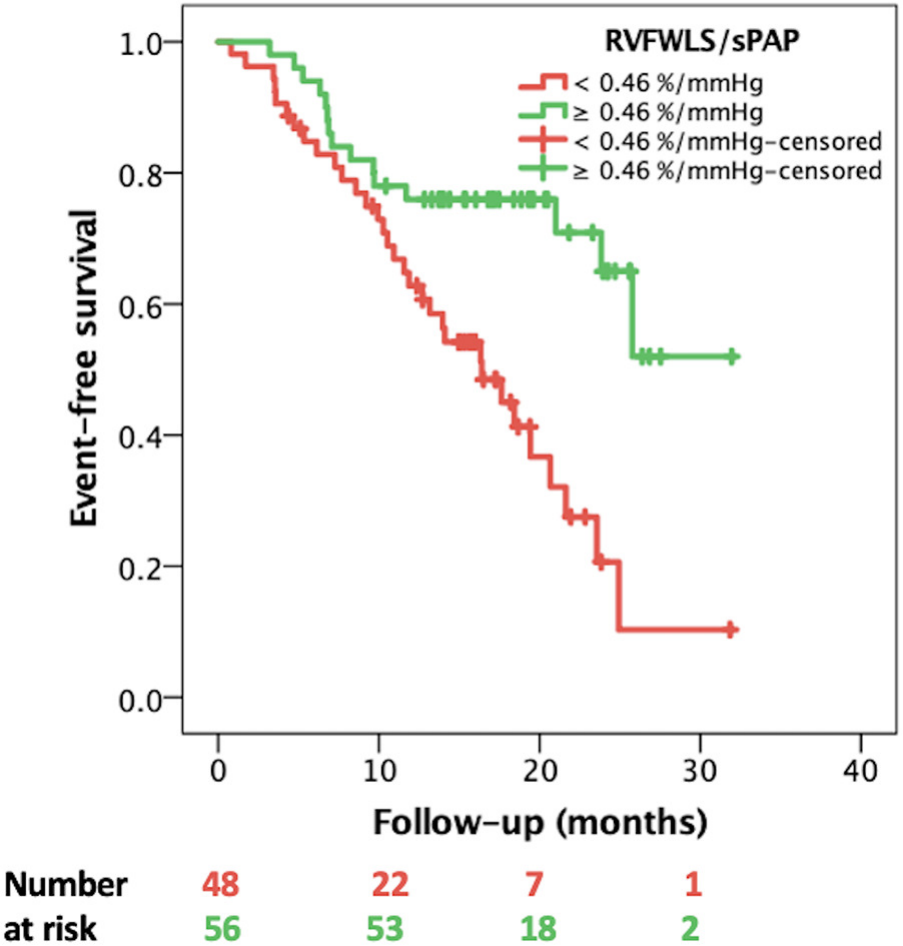
Kaplan–Meier event-free survival curves stratified by RVFWLS/sPAP < and ≥ 0.46%/mmHg. RVFWLS, right ventricular free wall longitudinal strain; sPAP, systolic pulmonary artery pressure.

In univariate Cox regression analysis, the clinical variables significantly associated with the composite endpoint (Table 2) were NYHA class (HR 2.054, 95% CI: 1.433–2.924, *p* < 0.001), type 2 diabetes mellitus (HR 1.368, 95% CI: 1.081–1.728, *p* = 0.008), advanced (4 and 5) chronic kidney disease stage (HR 3.141, 95% CI: 1.574–6.269, *p* = 0.001). In addition, several echocardiographic variables demonstrated a significant association with the composite endpoint, including all five RVPA coupling surrogates (Table 2). Accordingly, the prognostic power of the non-invasive, echocardiographic indices of RVPA coupling was tested in a ROC curve analysis that revealed that RVFWLS/sPAP had the highest area under the curve (AUC) of 0.730 (95% CI: 0.603–0.856 Figure 2), significantly outperforming the index with the second highest AUC, RVFAC/sPAP (0.684, 95% CI = 0.552–0.817). with a Z-statistic of 2.723, and a *p* value of 0.004 from the DeLong test, The optimal cut-off value for RVFWLS/sPAP determined using Youden's J index was 0.46%/mmHg (in absolute values), which provided a sensitivity of 71% and a specificity of 69% for predicting the composite endpoint. Kaplan–Meier survival analysis stratified by this cut-off demonstrated a significant difference in event-free survival between patients with an RVFWLS/sPAP above and below 0.46%/mmHg (log-rank *p* = 0.001, Figure 3).

**Table 2:** Univariable Cox regression analysis for the composite endpoint.

Parameter	HR [95% CI]	*P* value
Age	0.981 [0.963–1.000]	0.055
Sex	1.658 [0.799–3.441]	0.175
NYHA class	2.054 [1.443–2.924]	<0.001
Type 2 Diabetes Mellitus	1.368 [1.081–1.728]	0.008
Arterial hypertension	0.958 [0.535–1.714]	0.885
Dyslipidemia	1.370 [0.760–2.470]	0.294
Coronary artery disease	0.742 [0.412–1.336]	0.320
Chronic kidney disease stage 4–5	3.141 [1.574–6.269]	0.001
ACEi/ARNI	0.947 [0.450–1.993]	0.886
Betablockers	1.716 [0.529–5.564]	0.368
Spironolactone	0.835 [0.451–1.544]	0.564
Mitral regurgitation grade	1.783 [1.272–2.498]	0.001
Tricuspid regurgitation grade	1.376 [1.005–1.886]	0.047
Tricuspid regurgitation more than mild	1.652 [0.915–2.981]	0.092
LA maximum volume index	1.001 [0.984–1.018]	0.924
LA reservoir strain	0.958 [0.911–1.008]	0.097
LV global longitudinal strain	0.898 [0.817–0.987]	0.025
LV EDV index	1.008 [1.002–1.015]	0.013
LV ESV index	1.009 [1.001–1.016]	0.027
LV SV index	1.024 [0.993–1.055]	0.128
LV EF	0.986 [0.951–1.023]	0.457
sPAP	1.016 [1.003–1.030]	0.017
RV end-diastolic area index	1.173 [1.053–1.307]	0.004
RV end-systolic area index	1.161 [1.033–1.305]	0.012
RV FAC	0.971 [0.944–0.999]	0.044
TAPSE	0.937 [0.879–0.998]	0.044
RV FWLS	0.920 [0.877–0.996]	0.001
RV EDV index	1.006 [0.993–1.020]	0.346
RV ESV index	1.013 [0.994–1.032]	0.192
RV EF	0.955 [0.929–0.982]	0.001
RA maximum volume index	1.021 [1.006–1.037]	0.007
RA reservoir strain	0.981 [0.956–1.007]	0.145
RVEF/sPAP	0.564 [0.363–0.877]	0.011
RVFWLS/sPAP	0.162 [0.052–0.499]	0.002
RVFAC/sPAP	0.556 [0.329–0.941]	0.029
TAPSE/sPAP	0.291 [0.097–0.874]	0.028
RVSV/ESV	0.385 [0.151–0.980]	0.045

CI, confidence interval; HR, hazard ratio; other abbreviations as in Table 1.

In the multivariable Cox regression model (Table 3), after adjusting for confounders and choosing the variables based on their significance in univariate analysis, ROC analysis, and clinical relevance, the only two independent predictors of adverse outcomes remained type 2 diabetes mellitus (HR 1.464, 95% CI: 1.093–1.959, *p* = 0.010), and RVFWLS/sPAP (HR 0.110, 95% CI: 0.012–0.992, *p* = 0.049). Finally, adding RVFWLS/sPAP to the baseline model including clinical and echocardiographic data (namely type 2 diabetes mellitus, NYHA class, stage 4 or 5 chronic kidney disease, MR and TR grade, LV global longitudinal strain, LV EDV index, sPAP, and RA maximum volume index, variables included in the multivariable Cox regression analysis reported in Table 3), provided incremental prognostic value (*χ*^2^ 34.597 vs. 31.653, *p* = 0.025). However, at univariable logistic regression analysis, RVFWLS/sPAP was not predictive of all-cause mortality alone (odds ratio = 0.157, 95% CI = 0.024–1.034, *p* = 0.054).

**Table 3:** Multivariable Cox regression analysis for the composite endpoint.

Parameter	HR [95% CI]	*P* value
Type 2 Diabetes Mellitus	1.464 [1.093–1.959]	0.010
NYHA class	1.435 [0.983–2.094]	0.061
Chronic kidney disease stage 4–5	1.356 [0.564–3.261]	0.496
Mitral regurgitation grade	1.407 [0.868–2.281]	0.166
Tricuspid regurgitation grade	0.638 [0.383–1.062]	0.084
LV global longitudinal strain	1.027 [0.907–1.163]	0.673
LV EDV index	1.004 [0.996–1.013]	0.313
sPAP	0.991 [0.970–1.012]	0.391
RA maximum volume index	0.998 [0.977–1.020]	0.863
RVFWLS/sPAP	0.110 [0.012–0.992]	0.049

Abbreviations as in Tables 1, 2.

The advanced echocardiographic measurements of RV volumes and function by 3DE and STE showed excellent reproducibility. For the first measurement, RV EDV by 3DE had a CV of 0.41, RV ESV by 3DE a CV of 0.42, and RVFWLS by STE a CV of 0.40. Intra-observer reproducibility showed ICC values of 0.965 and CVs of 0.43 for RV EDV, 0.973 and 0.45 for RV ESV, and 0.99 and 0.42 for RVFWLS. Finally, the inter-observer reproducibility had ICC values of 0.988 and CVs of 0.44 for RV EDV, 0.975 and 0.45 for RV ESV, and 0.989 and 0.41 for RVFWLS, respectively.

## 4 Discussion

Our study demonstrates that a reduced RVFWLS/sPAP ratio is a powerful, independent predictor of adverse outcomes—specifically, rehospitalization for HF and all-cause mortality—in patients with HFrEF and V-sMR, even when adjusted for other clinical and echocardiographic confounders, with an optimal cut-off value of 0.46%/mmHg.

### 4.1 Comparison with conventional indices and prior studies

Until recently, the evaluation of RV function for the risk stratification of cardiac patients was largely neglected. Lately, RV dysfunction has emerged as an important predictor of patient morbidity and mortality in cardiovascular diseases (6, 23–26). However, the routinely performed, two-dimensional assessment of RV size or function has significant limitations. TAPSE is a load-dependent measure of RV function, susceptible to external interactions. Moreover, it can be affected by various factors that may lead to the underestimation of RV function, such as suboptimal image quality, misalignment of the cursor along the RV free-wall longitudinal axis, and cannot be used for RV function assessment after cardiac surgery. RV FAC is highly unlikely to accurately represent the complex three-dimensional geometry and crescentic shape of the RV, therefore it implies geometric assumptions for volumetric calculations, and does not take into consideration the contribution of the RV outflow to its pump function. Consequently, cardiac magnetic resonance (CMR) has become the gold-standard technique for the calculation of RV size and function (27). However, the true RV intrinsic performance, which has been extensively demonstrated to be closely related to afterload, is accurately represented not even by the CMR-derived RVEF (28–31). Moreover pulmonary artery pressures or pulmonary vascular resistance obtained by right heart catheterization (RHC) or estimated by Doppler echocardiography do not take into account the hydraulic component of RV afterload, that is arterial elastance (Ea), but only the steady one (32). RV end-systolic elastance (Ees), a load-independent parameter of RV function, and Ea, a measure of RV afterload by RHC derived pressure-volume loops represent the gold standard indices of ventricular-arterial function (33). RV-PA coupling expressed as Ees/Ea is the most accurate representation of RV systolic function in relation to afterload (33). However, RV evaluation by CMR, and RV pressure-volume loops by RHCcannot be routinely performed in every patient. Therefore, the new indices of RV function—RVFWLS by STE, or RVEF by 3DE might overcome the limitations of TAPSE or FAC by 2DE, the unavailability of CMR. However, they still exibit load-dependency, and, accordingly, echocardiographic RVPA coupling estimates expressed as the ratio between RV functional parameters and Doppler-derived sPAP have been proposed as valuable surrogate for invasively measured RVPA coupling in various clinical conditions, and could overcome the limitations of RHC (34).

TAPSE/sPAP has been widely used in the prognostic evaluation of cardiac patients, and has been validated against RHC-derived RVPA coupling (35). TAPSE/sPAP has been shown to predict the outcome in various cardiovascular conditions (pulmonary arterial hypertension, secondary TR (36), HF (29), in patients in the intensive care unit (37), or in patients undergoing MitraClip (15, 38). However, neither TAPSE/sPAP, nor FAC/sPAP demonstrated their risk stratification value in our cohort of patients.

Our findings extend prior work by incorporating RVFWLS—a parameter derived from myocardial deformation imaging that reflects subtle alterations in contractility. In contrast to TAPSE, RVFWLS provides a more nuanced and comprehensive assessment of RV function, which appears to translate into superior risk stratification. Interestingly, in our cohort of patients, RVPA coupling calculated as RVFWLS/sPAP was the best parameter for outcome prediction, with superior prognostic value even to RVEF/sPAP at ROC analysis. These findings likely reflect its greater sensitivity to early contractile dysfunction, and its reduced reliance on RV geometry and loading conditions (39), as we have previously demonstrated that patients with dilated cardiomyopathy and without clinical RV failure already exhibit reduced RVFWLS values compared to controls (40).

While an analysis from the COAPT trial (41) that focused exclusively on patients with severe secondary MR demonstrated that advanced RVPA uncoupling (RVFWLS/RVSP ≤0.5%/mmHg) robustly predicts adverse outcomes, our study expands on these findings by evaluating a broader HFrEF cohort that includes patients with varying degrees of MR—even those with mild MR, which has been shown to worsen prognosis, as even mild MR may adversely affect outcomes in patients with HFrEF (1). Interestingly, while the COAPT trial (41) reported a higher sensitivity (79%) for outcome prediction for RVFWLS/sPAP, the specificity was considerably lower (39.5%), potentially due to differences in the patient populations and methodological approaches. Our study, with a broader cohort of V-sMR severity, showed a more balanced sensitivity and specificity (71% and 69% respectively), highlighting that the predictive power of RVPA uncoupling can vary depending on the severity of MR and clinical context. These differences underscore the importance of considering RVPA coupling in a range of clinical scenarios, including patients with less severe MR, in order to improve risk stratification and improve patient management.

Finally, in our cohort of patients, the reliability of the RVFWLS/sPAP ratio is further supported by the predominance of patients with mild TR. Since TR can lead to an underestimation of sPAP by Doppler echocardiography due to rapid pressure equalization between the RV and the RA, this minimizes the risk of underestimation of sPAP and ensures that our measurement of RVPA coupling accurately reflects true hemodynamic conditions.

### 4.2 Pathophysiological and clinical considerations

The pathophysiology underlying our observations is complex. In HFrEF with sMR, LV remodeling elevates LA pressures and, subsequently, pulmonary arterial pressures. The RV must then adapt to a dual challenge: an increased afterload combined with volume overload. The RVFWLS/sPAP ratio effectively quantifies this interplay by coupling an index of intrinsic myocardial deformation with the degree of pulmonary hypertension. A lower ratio reflects a scenario where the RV is unable to adequately compensate for the increased load—a finding that has been consistently associated with adverse remodeling and poor clinical outcomes. However, in the majority of previous studies, RVPA coupling has been assessed as TAPSE/sPAP (42, 43). Our findings demonstrate that the RVFWLS/sPAP ratio is a robust, independent predictor of adverse outcomes, outperforming both traditional indices such as TAPSE/sPAP, RV FAC/sPAP, as well as novel ones, such as RVEF/sPAP and RV SV/ESV in our cohort of patients with HFrEF and V-sMR. However, from a pathophysiological point of view, the most accurate echocardiographic representation of RVPA coupling derived from RHC as surrogate of the ratio between Ees/EA remains the RVSV/ESV ratio. It has been used as an index of RVPA coupling in patients with HF and left-sided valve disease (44), and has been invasively validated against Ees/Ea (12). Yet, in our cohort of patients, the prognostic value of RVFWLS/sPAP outperformed the one of RVSV/ESV. Incorporating the RVFWLS/sPAP ratio into routine echocardiographic evaluations may significantly enhance risk stratification in HFrEF patients with V-sMR. Our results suggest that patients with RVPA uncoupling, as reflected by a low RVFWLS/sPAP ratio, could be identified earlier as high-risk, thereby prompting more aggressive or targeted therapeutic interventions. Nonetheless, it is important to note that the distribution of V-sMR severity in our cohort was skewed toward mild V-sMR (54% of cases), and this could limit the generalizability of our findings, particularly in populations with a higher prevalence of more severe V-sMR.

### 4.3 Future directions and limitations

While our findings are promising, several limitations warrant consideration. The retrospective design and modest sample size may limit the generalizability of our results. We did not stratify patients by HFrEF phenotype, and this could influence the prognostic value of echocardiographic RVPA coupling indices. Moreover, variability in strain imaging and reliance on operator expertise could impact the reproducibility of the RVFWLS measurement. Future prospective, multicenter studies with standardized imaging protocols are needed to validate the routine clinical use of the RVFWLS/sPAP ratio. Additionally, exploring serial changes in this ratio could provide valuable insights into disease progression and treatment response.

## 5 Conclusion

In summary, our study highlights the clinical value of the RVFWLS/sPAP ratio as a robust, independent prognostic marker in patients with V-sMR and HFrEF. By integrating a sensitive measure of RV contractility with the degree of afterload, this novel index offers incremental prognostic information beyond that provided by conventional parameters.

## Data Availability

The raw data supporting the conclusions of this article will be made available by the authors, without undue reservation.
